# P149: Production and use of an alcohol-based handrub for hand hygiene in the point G University Hospital of Bamako Mali

**DOI:** 10.1186/2047-2994-2-S1-P149

**Published:** 2013-06-20

**Authors:** L Bengaly, A Benedetta, L Diallo, AT Traore, ZH Harouna, M-N Chraiti, P Bonnabry

**Affiliations:** 1 University Hospital of Point G, Bamako, Mali; 2 Hôpitaux Universitaire de Genève, Genève, Switzerland

## Introduction

Heath care-associated infections constitute a public health problem. Hand hygiene is the most efficient measure for prevention and alcohol-based handrub (ABHR) is considered as the optimal measure. In setting the first World Challenge for the Patients Safety, the Point G University Hospital (PGUH) was one of the pilot sites of the OMS to implement the multimodal strategy of hand hygiene promotion. ABHR local production was initiated in this setting.

## Objectives

To present the results of ABHR local production for the period of January 2007 to June 2010.

## Methods

The chosen formulation of ABHR contains 80% of ethanol, 1.45% of glycerol and 0.125% of hydrogen peroxide. The concentration of the ethanol has been controlled with alcoholmeter and the dosage of the hydrogen peroxide has been perfomed with the potassium iodine in acidic conditions and by titration with sodium thiosulfate. ABHR samples have been sent to the Geneva University Hospital for quality controls. The ABHR quantities used have been valued from bottles delivered in care units.

## Results

A total of 7000 100 ml-bottles have been produced and the production costs has been estimated to 0.29 $US per bottle. The average concentration of ethanol was 80.51% v/v (± 1.89) and the one of the hydrogen peroxide at 0.123% v/v (± 0.0076). Controls done to Geneva reaffirmed the conformity of component concentrations to specifications of formulation and confirmed the absence of microbial contamination. The amount of ABHR global consumption increased from 7.44 ml/patient-day in 2008 to 5.31 ml/ patient-day in 2009, and reached 5.97 ml/ patient-day to the first semester of 2010.

## Conclusion

The principal difficulties were linked to the obtaining of bottles and caps for the ABHR bottling. Results of this survey showed that ABHR can be produced locally according to WHO recommendations with a good level of quality and stability. The example of PGUH can act as model for other hospitals in Mali and in other countries.

## Disclosure of interest

None declared

